# A successful intervention to improve conceptual knowledge of medical students who rely on memorization of disclosed items

**DOI:** 10.3389/fphys.2023.1258149

**Published:** 2023-08-30

**Authors:** Stefan Heber, Matthaeus Ch Grasl, Ivo Volf

**Affiliations:** ^1^ Centre for Physiology and Pharmacology, Institute of Physiology, Medical University of Vienna, Vienna, Austria; ^2^ Department of Otorhinolaryngology, Head and Neck Surgery, Medical University of Vienna, Vienna, Austria

**Keywords:** item preknowledge, assessment, item bank, learning strategies, learning outcome

## Abstract

**Background:** The mere memorization of isolated facts without the claim of integrating them is detrimental to the desired learning outcomes in medical education. The current study was conducted within an environment where items from summative assessments are regularly disclosed by the university and consequently collected into an item bank that is shared between students. Within this environment, we aimed to quantify 1) to which extent students use disclosed items for their preparation for the summative exam, 2) a putative mismatch between (isolated factual) knowledge regarding distinct questions from the item bank and conceptual knowledge, and 3) to which extent this mismatch can be ameliorated by a project aiming to steer student learning away from the memorization of isolated facts toward the acquisition of conceptual knowledge.

**Methods:** This steering project in the midst of the first semester consisted of the implementation of an oral exam based on selected learning objectives, preceded by two seminars. After their summative exam at the end of semester, 135 students performed a voluntary exam for study purposes. Here, authentic (i.e., presumably preknown) physiology questions taken from the item bank were used to assess students’ ability to 1) recognize the correct answer in a multiple choice (MC) question, 2) recall the answer (short answer), or 3) display conceptual knowledge closely corresponding to the question presented in the other formats. Additionally, students received a questionnaire addressing their learning habits and attitudes.

**Results:** The median reported percentage of learning time for the summative exam exclusively spent with this item bank was 80%. The results of the voluntary exam indicate that students frequently recognize and recall correct answers of included items without displaying knowledge of the underlying concept. Compared to recall of the correct answer, the probability of giving a correct answer regarding the corresponding basal physiologic concept was lower by 47 percentage points (*p* <0.001) for topics not included in the steering project. Regarding topics included in the steering project, this discrepancy was reduced to 25.5% (*p* <0.001).

**Conclusion:** The results of this study demonstrate the influence of disclosed items on student learning and learning outcomes and suggest that a carefully implemented assessment is able to improve conceptual knowledge in physiology.

## 1 Introduction

Assessment plays a central role in both the formal and hidden curriculum (McCoubrie, 2004). Proper assessment ensures that the intended educational goals are met and identifies students that lack the required competence.

Consequently, there has been a long lasting discussion regarding the characteristics of a “proper” assessment, especially concerning the applied test format. Severe criticism has been posed on multiple choice (MC) questions as they expose the test taker to the correct answer and might, therefore, test the memory recall of independent facts rather than the generation of the correct answer or application of knowledge (Vanderbilt et al., 2013).

Although it is currently accepted that the question content and quality is more important to the discussion of a “proper” assessment compared to the test format, assessment also represents the most important factor to steer student learning (Mattick, Dennis, and Bligh, 2004). Students prepare differently for examinations administered in different formats (Frederiksen, 1984; Cilliers et al., 2010; Cilliers et al., 2012), and assessments that rely on the recall of facts drive student learning to a surface-oriented approach (Mattick and Knight, 2007). Within such a surface-oriented approach, the learner focuses on the simple memorization of isolated parts of the subject matter without the aim of integrating them. Especially, undergraduate students in the early phases of their studies are prone to using this approach (Mattick et al., 2004) that has been characterized as the acquisition of “fragmented knowledge” or “bits of information.” In contrast, conceptual knowledge has been defined by “how bits of information are interconnected and interrelated” (Bloom et al., 1956; Anderson et al., 2001).

While related research has focused on the influence of the question type and question quality on learning outcomes, a factor presumably central to the learning behavior of medical students has received little attention: the availability of unofficial item banks. If students are aware of an item bank that holds a substantial proportion of questions relevant to an assessment, an impact on student learning will be unavoidable. While disclosed questions might define a narrow topic and thereby serve as a starting point for diverse self-directed learning activities, availability of correct answers within such item banks might favor the memorization of these answers. Thereby, the acquisition of isolated factual knowledge might preferably apply to MC questions, irrespective of the question quality.

Apparently, rote memorization of facts and acquisition of isolated factual knowledge are detrimental to the desired learning outcomes in medical education. Therefore, it is crucial to analyze the learning environment on aspects that might negatively affect student learning and learning outcomes and set measures to assure that the intended goals of the curriculum are indeed met.

The current study was conducted with first-year medical students within an environment where items from summative assessments are disclosed by the university (MC question stem plus correct answers) and are, in turn, collected by students in a student-administered item bank. Consequently, data included in this item bank are comprehensive and lecturers perceive that the memorization of these items shows a detrimental influence on student learning, resulting in a lack of a broader understanding of physiologic concepts.

Within the current study, we quantified to which extent disclosed items are used by students for their preparation for the summative MC-based exam. To this end, the study included a cross-sectional part in the form of a questionnaire. This defined the environment, in which the interventional trial was performed, which had two aims.

Aim 1 was to test the assumption that, in the described environment, isolated factual knowledge shows dominance over the knowledge of basic concepts in physiology. We assumed a greater probability for students to correctly answer authentic questions from the item bank (be it the recognition or recall of the correct answer) compared to questions dealing with a very closely related aspect of the very same physiologic concept that was central to the question from the item bank.

Aim 2 was to test an intervention, aiming to steer student learning away from memorization toward a more holistic approach. Within this steering project (SP), students were provided with closely defined learning objectives. The implementation of two seminars allowed students to discuss unclear concepts, and this was followed by a compulsory oral exam, also intended to give feedback to students regarding their performance to assist the learning process.

Thereby, the intervention exposed students to learning objectives (SP_objectives_^+^) relevant to both the oral exam and the summative exam at the end of the semester, while other objectives included within the same learning module were (in terms of the assessment) exclusively relevant to the summative MC exam at the end of the semester but were not included in the learning objectives of the SP (SP_objectives_^−^).

For study purposes, 135 students were assessed in a voluntary exam (VEX) four days after the summative MC exam to investigate whether the intervention improved conceptual knowledge. Therefore, physiology questions included in the item bank were categorized to be part of SP_objectives_^+^ or SP_objectives_^−^ and were randomly assigned for inclusion in the VEX.

The hypothesis corresponding to aim 2 was that student performance on authentic questions from the item bank was independent from the fact if these questions were covered by the learning objectives of the steering project. In contrast, we assumed a higher probability of a correct answer for questions based on conceptual knowledge in favor of SP_objectives_^+^ compared to SP_objectives_^−^.

## 2 Materials and methods

### 2.1 Study overview

The first semester of the integrated medical curriculum at the Medical University of Vienna consists of three modules (“Healthy and sick people”—3 weeks, “The human body”—6 weeks, and “From molecules to cells”—6 weeks). An MC-based exam at the end of the semester that includes the contents of the three modules represents the first summative assessment for students. After each summative MC exam, questions together with the correct answer are disclosed to students, who collect this information in an item bank that is shared between students. As the summative MC exam shows a relatively high percentage of reused questions, student preparation for the summative MC exam shows a strong focus on isolated information included within the item bank. To interfere with the consequences of this learning behavior, we implemented a steering project with the intention of affecting student learning behavior and learning outcome away from memorizing isolated facts toward the acquisition of conceptual knowledge and a comprehension of concepts. The SP was implemented for 744 students within the second module of the first semester.

**FIGURE 1:**
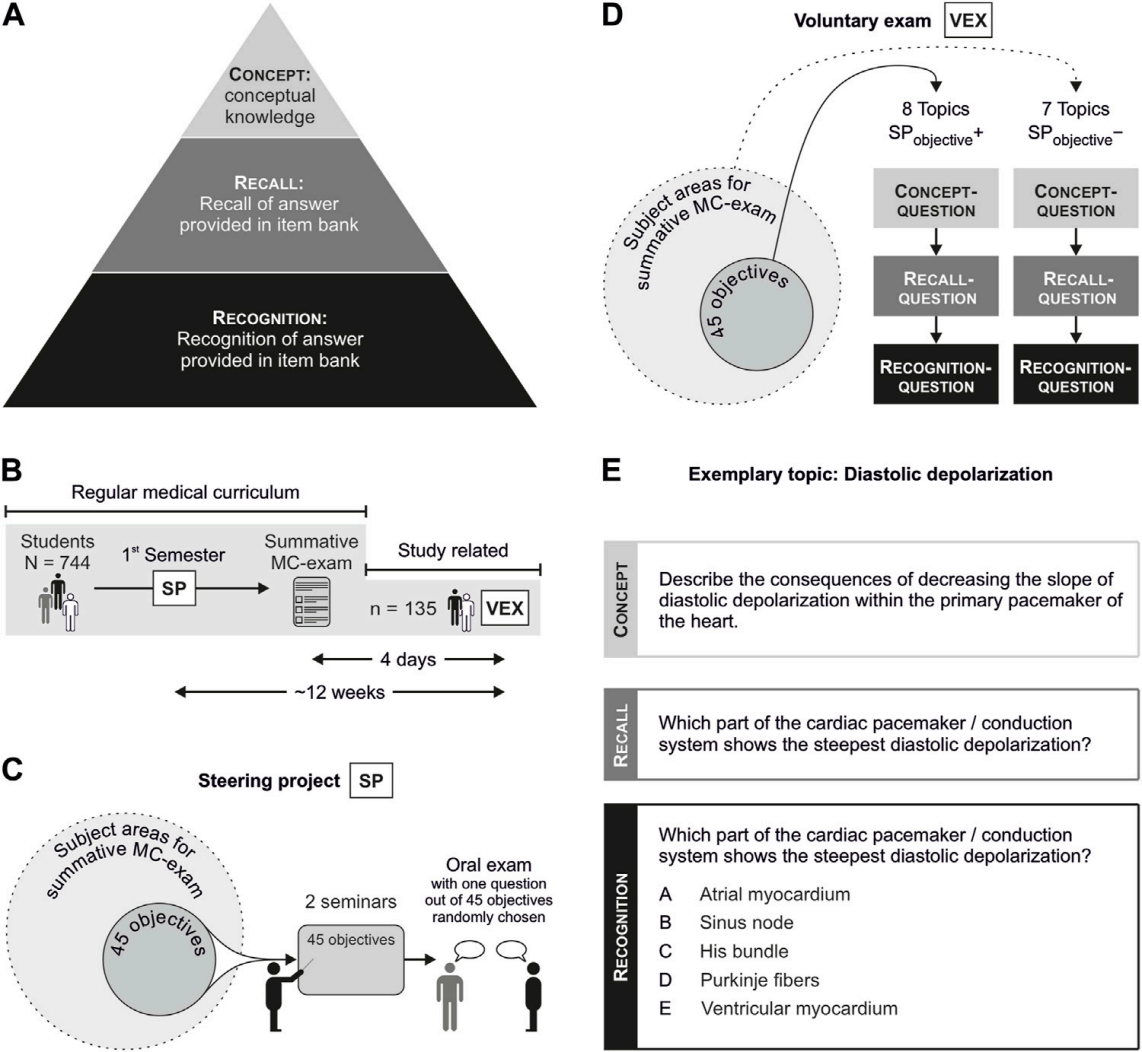
Overview and selected parts of the trial design: **(A)** definition of competence levels as applied for the VEX; **(B)** regular medical curriculum, the SP, and the VEX; **(C)** steering project: graphical scheme; **(D)** VEX: graphical scheme; **(E)** example of questions on the topic of diastolic depolarization as applied for the VEX, representing different competence levels. Abbreviations: VEX, voluntary exam; SP, steering project; MC, multiple choice.

Four days after the summative MC exam at the end of the semester (and ∼12 weeks after the SP), 135 voluntary students were tested on physiological topics in the VEX. The basic scheme is shown in Figures 1A, B.

#### 2.1.1 Steering project for all 744 students in the first year of the regular medical curriculum

A total of 45 learning objectives (SP_objectives_^+^) were defined within the subject areas of neurophysiology, muscle and motor system/function, hormonal system, renal system, ventilatory system, gastrointestinal system, and the cardiac and circulatory system (for a graphical overview, see Figure 1C). Three experienced physiologists, responsible for teaching the physiological content within module 2, agreed upon the selection of the learning objectives that intentionally only covered a part of the respective subject areas. Learning objectives were disclosed to students in the week before module 2 started, and students were informed that the newly implemented oral assessment would focus on the comprehension of physiological concepts strictly related to SP_objectives_^+^. Moreover, two mandatory seminars (two academic hours each) preceding the oral assessment were implemented to allow students to discuss unclear concepts with the most experienced lecturers that were involved in physiological teaching within this module (including those who defined the learning objectives of the SP and agreed on question items and acceptable answers within the VEX).

The central part of the SP was a mandatory oral exam that was strictly based on the disclosed learning objectives. It was organized for 744 students on three consecutive days. As a consequence of the large number of students, the approach to focus on conceptual knowledge and the number of available examiners, the time frame available only allowed for one physiologic question that was randomly assigned to each student. As the implementation of the SP was primarily intended to impact student learning (behavior) and not to achieve reliable test results, this limitation was considered acceptable. For those students who failed this exam, two additional dates were scheduled. Passing rates at these occasions were 87%, 73%, and 54% of participating students (cumulative rates: 87%, 97%, and 98%, respectively). In summary, 12 students failed to pass the exam due to a negative test result or absence.

#### 2.1.2 Voluntary exam for 135 students who participated in the present study

The purpose of the VEX was to assess the effectiveness of the steering project. It was performed with 135 voluntary students (see also Section 2.5 Student recruitment for the VEX) four days after the summative MC exam at the end of the semester (and ∼12 weeks after the SP). The VEX represented a written assessment, consisting of questions on topics that were all relevant to both module 2 and the summative MC exam, but were only in part covered by the learning objectives of the SP (i.e., the VEX consisted of SP_objectives_^+^ and SP_objectives_^−^, see Figure 1D).

Physiology questions from the disclosed item bank were categorized to either be a part or not be a part of SP_objectives_^+^. Eight random questions each were chosen, and by mistake, one question within SP_objectives_^−^ was not included in the printout of the VEX, resulting in a final number of seven SP_objectives_^−^ and eight SP_objectives_^+^ questions.

Each topic was tested by different questions (example shown in Figure 1E; a complete list provided in Supplementary Materials), with each question covering one of the three competence levels (CLs; concept depicted in Figure 1A): CL_recognition_ (defined by MC questions that were taken from students’ item bank, including five answer options), CL_recall_ (open question regarding question stem of CL_recognition_), and CL_concept_ (open question regarding a basal concept underlying the question from the item bank).

Thereby, CLs were intended to discriminate within the knowledge domain according to Bloom’s taxonomy between factual knowledge, “bits of information,” and conceptual knowledge, “how bits of information are interconnected and interrelated” (Bloom et al., 1956; Anderson et al., 2001). As the student item bank only included correct answers without distractors, MC questions were counter-checked with the university’s official internal item bank to present the questions with the original distractors.

Three experienced physiologists—all lecturers engaged in module 2—agreed that questions regarding CL_concept_ were very closely related to CL_recognition_ and further covered a central learning objective of this module. The same persons agreed upon correct answers to CL_concept_ (and CL_recall_, as the given answers might extend the distractors presented in CL_recognition_). Special care was taken so that the concept that was assessed in CL_concept_ was not directly addressed by any question within the item bank.

The VEX was paper-based, and students had 1 h for answering the provided questions, which was sufficient time for all participating students to complete it. Students received the respective questions in the following sequence: 1) CL_concept_, 2) CL_recall_, and 3) CL_recognition_ (see Figures 1D,E). When students had completed the questions of the respective CL, answer sheets were collected, preventing any subsequent adaptations of responses. Thereafter, questionnaires of the next CL were distributed.

After the students had completed the VEX, they were asked to respond to 13 survey items (paper-based), including the clarity of the provided learning objectives for the SP, their learning behavior, and the perceived effects of the SP (all: 6-point Likert scale). Additionally, they were asked about the time point when they began to deal with the provided learning objectives (7-point Likert scale), how many attempts they needed to pass the oral exam (free input), if they participated in the summative MC exam (yes/no), and their impression of the SP (free input).

### 2.2 Outcome

The outcome was whether a question was answered correctly or not and was assessed at the end of the study in the course of the VEX.

### 2.3 Sample size

Two sample size calculations were performed, one for the number of topics that had to be randomized in each group and one for the students that had to be included to determine the probability of a correct answer for a question with adequate precision. Sample size calculations were performed using nQuery 8.

#### 2.3.1 Sample size considerations regarding the number of topics

This sample size calculation was based on a simplification of the primary hypothesis, namely, that the mean of the answer probabilities at CL_concept_ differs between SP_objectives_^+^ and SP_objectives_^−^. We defined a difference of the arithmetic mean correct answer probability of 16% as the relevant difference and expected the standard deviation within each group to be 10%. A sample size of 8 in each group was determined to have a power of 84.48% to detect a difference in means of 16, assuming that the common standard deviation is 10 using a two group *t*-test with a 5% two-sided significance level.

#### 2.3.2 Sample size considerations regarding the number of students

The lowest precision of a proportion is obtained when the percentage of correctly answered questions is exactly 50%. For a given number of cases, the precision increases (i.e., the 95% confidence interval narrows) as the percentage deviates from 50%. Therefore, the estimation of the necessary number of students was based on the “worst case” assumption of 50% correct answers. We aimed for a 95% confidence interval to have a width of 20 percentage points at the maximum. The calculation showed that when the sample size is 111, a two-sided 95% confidence interval for a single proportion will range from 0.404 to 0.596 when the sample proportion is 0.5. The confidence interval was calculated using the Clopper–Pearson exact method.

### 2.4 Statistical methods

The hypothesis was that the probability of a correct answer to a question is higher for SP_objectives_^+^ compared to SP_objectives_^−^ and that this difference is greater for questions targeting CL_concept_ compared to questions included in the item bank.

To test this two-way interaction hypothesis, a generalized linear mixed model was applied.

The binary target variable was “answer” with the levels “correct” and “incorrect.” Thus, a model with a binomial distribution and a logit link was used.

The study design was represented in the model given as follows. The “competence level” with the three levels “concept,” “recall,” and “recognition” was used as a fixed factor, as this factor was part of the interaction hypothesis.

Whether a topic was part of the objectives for the steering project or not was also represented by another fixed factor, namely, “SP objective” with the levels “SP_objective_^+^” and “SP_objective_^−^.” A fixed factor was used because this factor was part of the interaction hypothesis SP_objective_ × competence level.

To take into account the dependency of the answers of the students, each student was used as one level of a random factor.

Furthermore, as there are more and less difficult topics, each topic was also used as a level of a random factor.

In addition, there were two topics whose associated questions regarding CL_concept_ had two variations. Therefore, “variation” with levels “1” and “2” was used as a random factor.

The degrees of freedom were calculated using the residual method.

The following contrasts were planned in case the interaction term was significant: 1) to compare the probability of a correct answer within each level of the “competence level” between “SP_objectives_^+^” and “SP_objectives_^−^” and 2) to compare competence levels within SP_objectives_^+^ and SP_objectives_^−^.

In the event that the interaction corresponding to the main hypothesis was not significant, it was planned to eliminate the interaction term from the model and interpret the main effects, again with appropriate pairwise comparisons.

#### 2.4.1 Exploratory analyses

To test the effect of continuous covariates, each was included separately in the existing model as a metric predictor. The interactions with the other predictors were tested first. If not significant, the interactions that included covariates were eliminated from the model, starting with the three-way interaction, until only the significant interactions remained or the covariate as the main effect remained. From the models including the non-significant interactions, the probabilities at each level of the fixed factors were estimated at different values of the covariate using IBM SPSS syntax CONTROL keyword in the EMMEANS subcommand in the GENLIN command.

Statistical analyses were conducted using IBM SPSS 27; graphs were created using GraphPad Prism 9.0. The *p* value ≤0.05 was considered significant.

### 2.5 Student recruitment for the VEX

Voluntary students were recruited for “feedback to the SP” by personal announcements in lecture halls and by mails from the Students’ Union at the beginning of the semester. The registration and participation in this “feedback” took place within the framework of a separate, independent course (621.000—Learning strategies in medical studies), which ensured an independent framework—also in terms of the study law.

### 2.6 Item bank

By the time of selecting and constructing questions for the VEX, the available item bank (AnkiWeb, 2020) consisted of 965 questions attributed to module 2 (the module relevant to the study described here).

## 3 Results

### 3.1 Self-reported learning activities and the perception of the SP

Within the general evaluation part of this study, the feedback of students was collected concerning their learning activities and their perception of the implementation of the SP.

**FIGURE 2:**
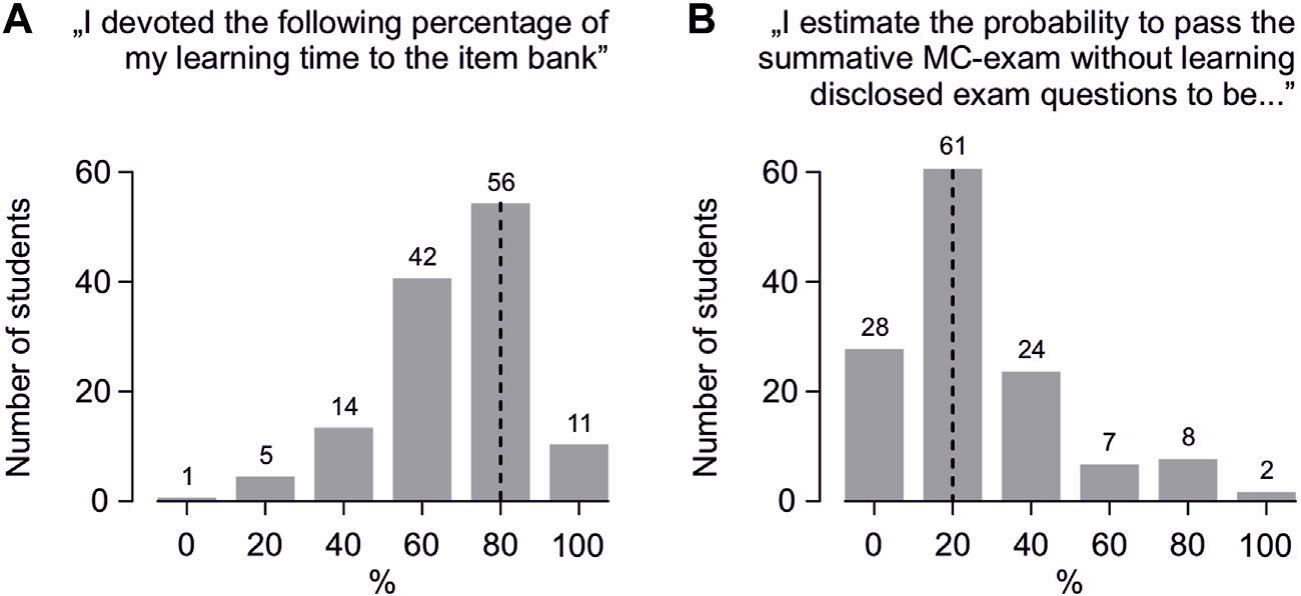
Student feedback regarding disclosed exam questions: **(A)** “What percentage of your learning time for the summative exam did you spend with learning disclosed exam questions?” **(B)** “In your opinion, what is the probability (in percent) of passing the exam without learning the disclosed exam questions?” Estimates were collected using a 6-point Likert scale (plus a “do not know” option). The feedback that was marked as “do not know” or that contained a number of response options different from one was cast as an invalid vote, resulting in a slight deviation from the total number of returned questionnaires (that was 135). The vertical dotted lines indicate the median.

On a 6-point Likert scale, the median reported percentage of learning time for the summative MC exam spent exclusively with questions from the item bank was 80% (Figure 2A). Regarding the perceived usefulness of these habits for the summative exam, the median estimated probability of passing this exam without learning the available questions was 20% (Figure 2B).

**FIGURE 3:**
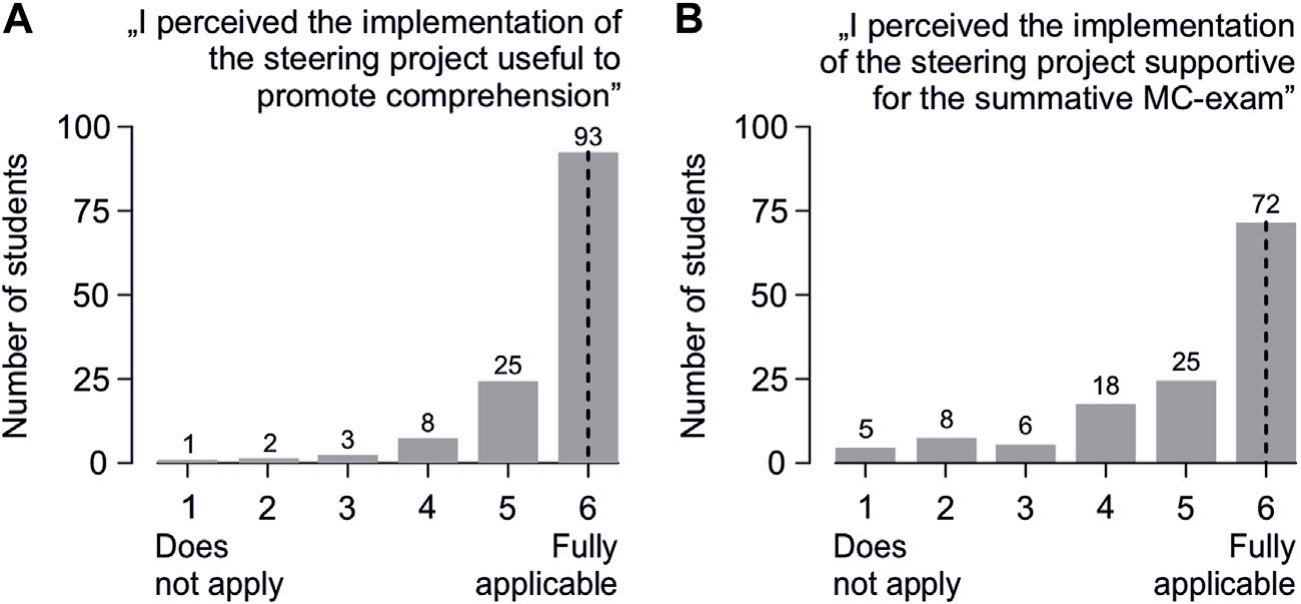
Student feedback regarding the implementation of the steering project: “I perceived the implementation of the steering project …” **(A)** “…helpful to promote comprehension of the content covered” and **(B)** “…supportive for learning for the summative MC exam”. Estimates were collected using a 6-point Likert scale (plus a “do not know” option). The feedback that was marked as “do not know” or contained a number of response options different from one was cast as an invalid vote, resulting in a slight deviation from the total number of returned questionnaires (that was 135). The vertical dotted lines indicate the median.

Regarding the student perception of the implemented steering project, the majority of students agreed with the statement that the implementation of this project was helpful in promoting comprehension of the covered topics (Figure 3A) and was supportive for learning for the summative MC exam (Figure 3B).

### 3.2 Student performance on different competence levels within SP_objective_^−^ topics

**FIGURE 4:**
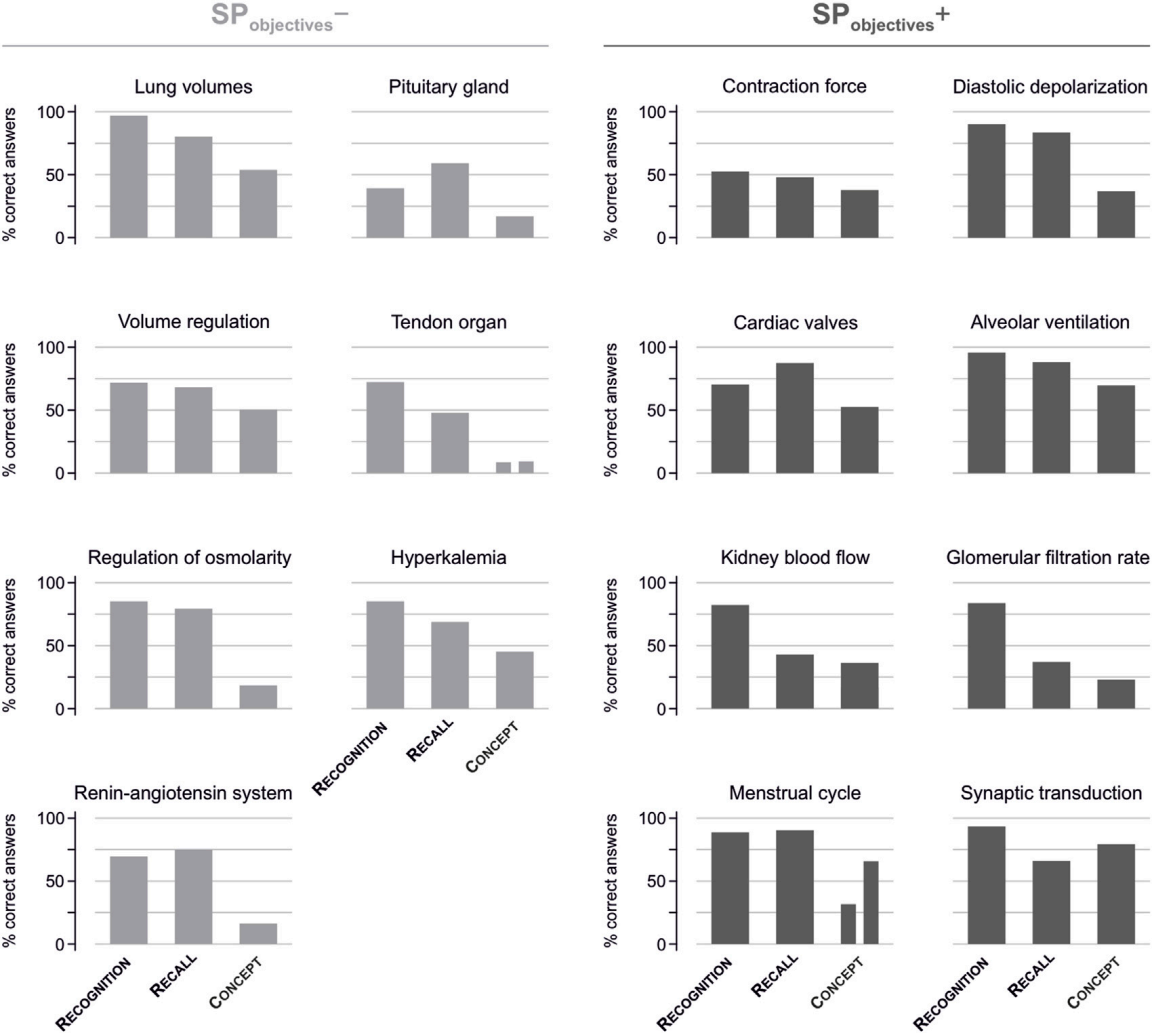
Correctly answered questions per topic and competence level. The percentage of correctly answered questions within each question topic and presentation per topic according to the competence level. Left: question topics regarding SP_objectives_^−^. Right: question topics regarding SP_objectives_^+^. It should be noted that the topics “Tendon organ” and “Menstrual cycle” were reflected by two distinct questions aiming to assess conceptual knowledge.

**FIGURE 5:**
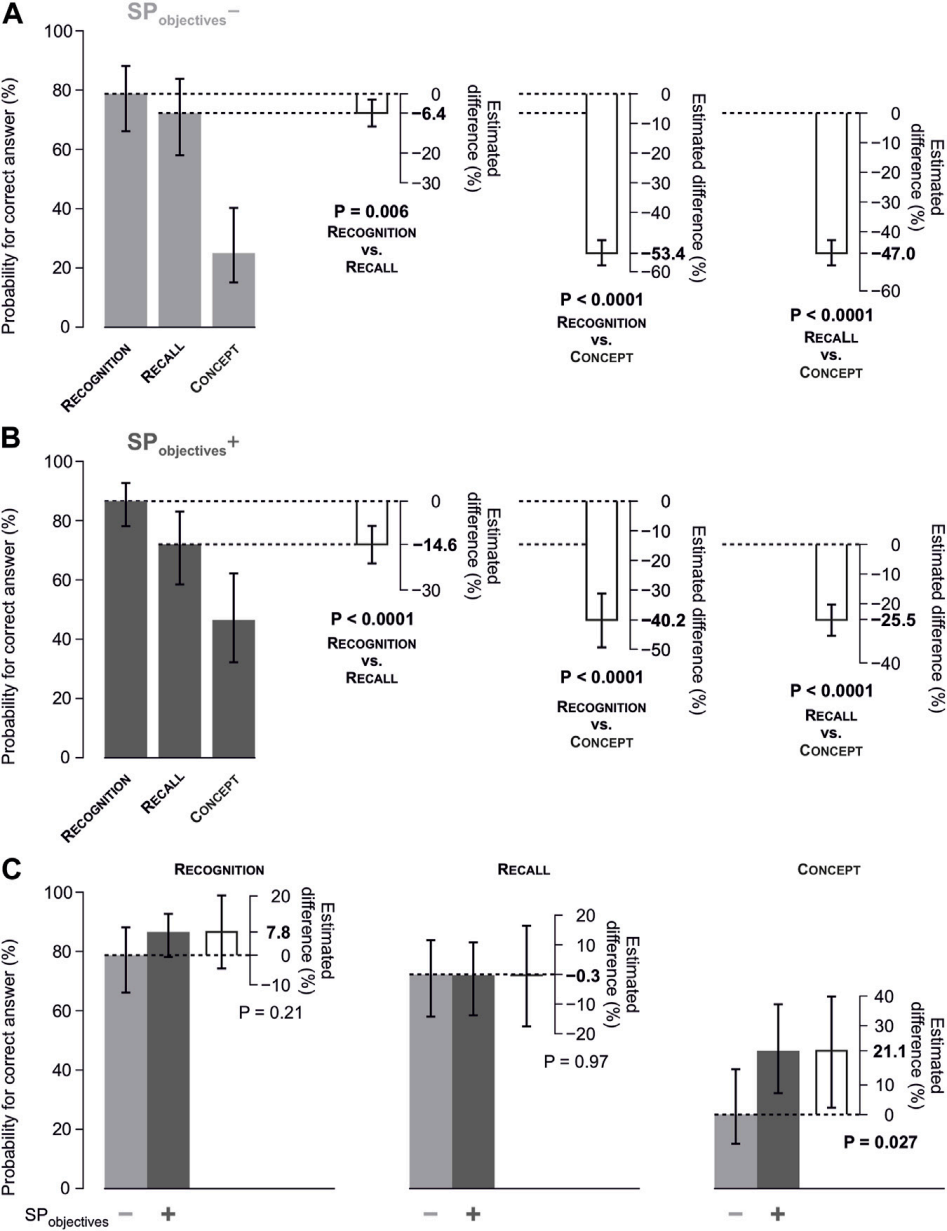
Estimated correct answer probabilities concerning questions per competence level. **(A)** Estimated probabilities with 95% confidence intervals concerning questions regarding SP_objectives_^−^, separately by competence levels CL_recognition_, CL_recall_, and CL_concept_. The plots on the right with white bars show the estimated difference in probabilities between the levels of competence with their corresponding *p* values. **(B)** Analogous plots as in (A) for questions regarding SP_objectives_^+^. **(C)** Comparison of the estimated correct answer probabilities between SP_objectives_^+^ and SP_objectives_^−^, separately by the competence level.

Within SP_objective_^−^ topics, the probability of a correct answer decreased statistically significantly and substantially from CL_recognition_ over CL_recall_ to CL_concept_. In particular, the probability for a correct answer was 79.3% (95% CI of 66.2–88.2) at CL_recognition_, 72.8% (58.0–83.9) at CL_recall_, and only 25.8% (15.2–40.3) at CL_concept_ (Figure 4, left; Figure 5A).

### 3.3 Student performance on different competence levels within SP_objective_^+^ topics

Within SP_objective_^+^ topics, a decrease of student performance over the respective competence levels was observed as well. The corresponding probabilities were 87.1% (78.2–92.7) at CL_recognition_, 72.5% (58.5–83.1) at CL_recall_, and 47.0% (95% CI 32.3–62.2) at CL_concept_ (Figure 4, right; Figure 5B).

### 3.4 Differences between SP_objective_^+^ and SP_objective_^−^ topics at CL_concept_

As depicted in Figure 5, the decrease from CL_recognition_ over CL_recall_ to CL_concept_ differed between topics assigned to SP_objective_^+^ and those assigned to SP_objective_^−^ (interaction “competence level” × “SP_objective_”; *p* <0.0001). Specifically, questions at CL_concept_ relating to SP_objectives_^+^ (correct answer probability 47.0%) were answered correctly with a higher probability by 21.1 percentage points (95% CI of 2.4–39.9; *p* = 0.027, Figure 5C) than those regarding SP_objectives_^−^ (correct answer probability 25.8%). There was no relevant difference between SP_objective_^+^ and SP_objective_^−^ topics at CL_recall_ and CL_recognition_.

Thus, data are in line with the hypothesis that the SP increases the probability for a correct answer in a competence level-dependent manner. The implementation of the SP was associated with an improvement of a student’s conceptual knowledge of topics included in the learning objectives of SP without affecting students’ ability to recognize or recall the correct answer to multiple choice questions or the respective question stem.

### 3.5 Relationship between item bank usage and performance in the VEX

**FIGURE 6:**
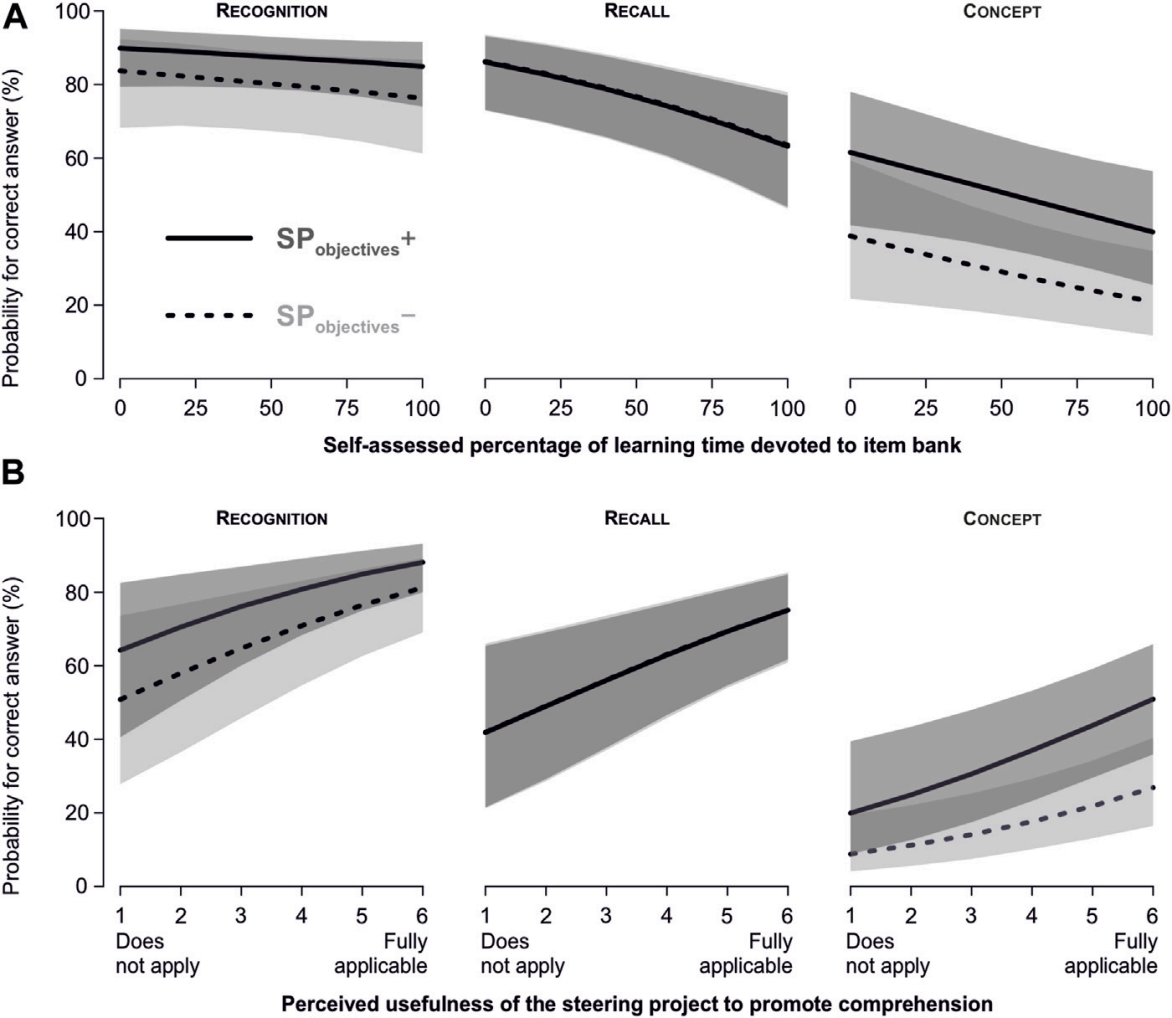
Relationship between correct answers vs. the time spent with disclosed questions and the perception of the SP. **(A)** Relationship between the amount of learning time spent with disclosed questions from the item bank and the probability for a correct answer in the VEX. **(B)** Relationship between the perception of whether the steering project supported comprehension and the probability for a correct answer in the VEX. Error bands are 95% confidence intervals.

The self-reported percentage of the total learning time spent with disclosed questions was inversely related to the performance in the VEX. The higher the reported proportion of learning time spent with questions from the item bank, the lower the probability for a correct answer (*p* = 0.015). This relationship was not significantly different between competence levels (*p* = 0.09, Figure 6A).

### 3.6 Relationship between the perception that the implementation of the SP enhanced content-related understanding and performance in the VEX

The perception of whether the SP enhanced content-related comprehension was positively related to VEX performance (*p* <0.001). This was not different between competence levels (*p* = 0.34, Figure 6B).

## 4 Discussion

This study was conducted with first-year medical students in an environment where students collect disclosed items of summative exams in a comprehensive item bank that is constantly updated and subsequently made available to all students.

The availability of this item bank clearly affects student learning, as the median reported percentage of learning time exclusively spent with disclosed items was 80%. Within this environment, it was appealing to use a subset of these disclosed questions in an attempt to get deeper insights into students’ learning outcome. The findings suggest that this item bank is used for the memorization of answers and thereby the acquisition of fragmented knowledge, rather than to serve as a starting point for diverse self-directed learning activities resulting in conceptual knowledge or comprehension.

This view is based on one of the main findings from this study: compared to a question (stem) taken from the item bank, students in the described environment showed a much lower probability for giving a correct answer regarding the basal physiologic concept of the same topic.

While an unknown (as was the case with CL_concept_) question is necessarily more difficult compared to a preknown question, the drop in the correct answer probability from CL_recognition_ to CL_concept_ by 53.4 percentage points in the absence of the SP was surprisingly large. This implies that many students were able to answer preknown questions without having deeper knowledge of the underlying concept. Notably, according to the consent of three experienced physiologists—all engaged in teaching within the relevant module—they agreed that questions regarding CL_concept_ were very closely related to CL_recognition_ and further covered a central learning objective of this module.

The memorization of question/answer pairs from an item bank would apparently represent a prime example for a surface approach of learning that is evidently detrimental to the desired learning outcomes in medical education. Even more so, if surface learning is seen essential for succeeding in an assessment, consequences on students’ general view on appropriate learning (strategies) can also be expected, thereby also affecting their personal approach to future learning (Struyven et al., 2005; Walke et al., 2014).

The second major finding of this study is that the topics covered by the implemented steering project were associated with improved learning outcomes, specifically at CL_concept_ compared to topics that were not covered. The SP was the major intervention to be tested in this study. It was expected to add a different learning stimulus and consisted of a mandatory oral exam that was strictly based on the defined learning objectives. Additionally, two seminars preceding this oral exam allowed students to discuss unclear concepts.

Our data are in line with the view that the steering project fulfills its intended purpose, namely, to steer student learning at the beginning of their studies and to provide scaffolding within this early phase of study where students have to make decisions about what (and how) to learn and where they appear to be especially prone to applying the surface approach (Mattick et al., 2004).

Student feedback indicates an exceptionally high level of agreement that this project has significantly improved their understanding of physiological concepts and that it supported their learning for the summative exam. Comments provided through free-text input (not shown in detail) revealed a high level of satisfaction, with the most frequently mentioned positive aspects being the guided steering of their learning process and the oral format of the SP exam.

The effect of the applied intervention observed in this study corresponds nearly to a doubling of the probability (from 25.8% to 47.0%) for a correct answer regarding physiological concepts. While we consider this improvement of conceptual knowledge to be impressive, it is also disappointing that the values resulting from the successful intervention are below 50%. Clearly, the effects of the item bank, consisting of “bits of information” rather than conceptual knowledge, on student learning for the summative exam are dominant. However, for the full interpretation of these data, it must be considered that conceptual knowledge was evaluated in the VEX four days after the summative exam, while the intervention had occurred 12 weeks earlier.

Another aspect worth mentioning is the modest, yet statistically significant decrease in the percentage of correct answers from CL_recognition_ to CL_recall_, both in SP_objectives_^+^ and SP_objectives_^−^. Although this is consistent with concurring findings obtained within the last decades that open questions are in general more difficult compared to the selected response format (Newble et al., 1979; Melovitz Vasan et al., 2018; Sam et al., 2019; Mehta et al., 2020), this was somewhat surprising for us, as we did not expect this behavior for preknown questions. Moreover, the student item bank only holds information regarding the correct answer(s), as distractors are not disclosed to the students. Therefore, the memorization of the correct answer would be expected to focus strictly on the available answer and not on pattern recognition.

The findings presented here support the viewpoint that the implementation of the steering project was successful and showed positive effects on student’s learning outcome in the field of physiology. However, the dominant effect of the existing item bank on students’ learning behavior requires further attention. It is comprehensible that the availability of questions from previous exams arouse the curiosity of students, but the focus they place on these questions will depend on the (perceived) authenticity of the questions and the assumed benefit that can be obtained from the available questions.

A random check of the available item bank revealed that the information collected there is highly accurate. This is not surprising, as questions are disclosed by the university to the students after each summative exam (in the form of a question stem and correct answers). The rationale for this disclosure lies in the attempt to avoid a legally required option for the exam inspection for more than 700 students and probably also includes the claim to give “feedback” to students. Consequently, students continuously transfer this “feedback” to the existing item bank, resulting in a comprehensive and continuously growing collection of items. Students’ perception of the assumed benefit that can be obtained from this collected information has been assessed within this study and explains their motivation to focus on the item bank. The information available there (question stem plus correct answer) evidently favors mere memorization and the acquisition of fragmented knowledge. The availability of a comprehensive collection of items that are accurate and further available to all students, as described here, allowed essential insights into the student learning behavior and the consequences resulting thereof. In environments where several item collections might exist that differ in content, accuracy, and availability for the individual student, the influence of the disclosed questions on student learning, and, consequently, a resulting mismatch between factual and conceptual knowledge, might not be as pronounced as that in the environment described here.

However, as both item reuse (Joncas et al., 2018) as well as a certain amount of content leakage is probably unavoidable and represents an unfortunate reality in medical education (McCoubrie, 2004; Joncas et al., 2018), we consider the findings from this study to be of general interest, as they show both the attractiveness of disclosed items for students and the resulting consequences on the learning outcome. In this respect, our data are in line with the published results regarding the motivation for and consequences of surface learning (Dinsmore and Alexander, 2012; Dolmans et al., 2016). Regarding the implementation of the steering project, our findings support previous evidence that students perceive assessments as a dominant motivator for learning (Drew, 2010) and that the application of different test formats influences student learning behavior (Newble and Jaeger, 1983).

As the assumed benefit for assessments steers students’ learning behavior, assessments should be carefully monitored for existing confounders and adapted where required to assure that the assessment is aligned with the goals of the learning process (Gerritsen-van Leeuwenkamp et al., 2019). Nevertheless, this monitoring for existing confounders should be performed continuously, as we have noticed after the second year of the implementation of the SP that students published elaborations of the learning objectives on anonymous webpages. Although this is notably detrimental to a desired deep learning approach, the memorization of such elaborations might bring more information to the individual student than the memorization of answers to MC questions, but this can be seen as the lesser of two evils.

The limitations that appear to the study presented here include the general problem that, despite all efforts to avoid this, it cannot be excluded that SP_objectives_^+^ and SP_objectives_^−^ differ in principle regarding the difficulty level of the respective concept. Additionally, the number of questions used in the VEX was limited, which is why the estimate for the difference at CL_concept_ has considerable uncertainty, as quantified by the 95% confidence interval. Furthermore, caution should be taken regarding the generalization of our findings. While we suspect that conceptual knowledge can generally be improved by efforts similar to those described here, the (im)balance between factual knowledge and conceptual knowledge resulting from the memorization of disclosed question/answer pairs might also depend on quality of MC questions. Although the consequence of memorizing high-quality MC questions on the learning outcome remains to be established, it is noteworthy that the item bank that formed the base of this study (and thereby reflected questions used in the summative exam) did not contain content-rich questions.

In conclusion, our results suggest that the availability of disclosed exam items, along with the expectation that these items are essential for succeeding in the upcoming summative exam, is associated with a learning outcome characterized by the acquisition of isolated factual knowledge and poor comprehension of the underlying physiological concept. Our findings suggest that, within such an environment, the implementation of appropriate measures targeting physiologic concepts rather than isolated factual knowledge can exert a positive effect on the learning outcome.

As a certain amount of item leakage is probably unavoidable and the ability to continuously generate new test questions within a given test format is limited, the results of this study suggest the importance of continuously challenging the student learning behavior. This might be achieved by implementing a variety of test formats and measures to address different forms of knowledge.

## Data Availability

The original contributions presented in the study are included in the article/Supplementary Material; further inquiries can be directed to the corresponding author.

## Abbreviations

CL, competence level; MC, multiple choice; SP, steering project; VEX, voluntary exam.
