# Supplementary material for: A successful intervention to improve conceptual knowledge of medical students who rely on memorization of disclosed items
Source: Front Physiol. 2023 Aug 30;14:1258149. doi: 10.3389/fphys.2023.1258149 (PMC10499193; doi:10.3389/fphys.2023.1258149)
Supplement: Supplementary file 1 [file DataSheet1.pdf]

frontiers

Supplementary Material

A SUCCESSFUL INTERVENTION TO IMPROVE CONCEPTUAL KNOWLEDGE OF MEDICAL STUDENTS WHO RELY ON MEMORIZATION OF DISCLOSED ITEMS

Stefan Heber[1], Matthaeus Ch Grasl[2], Ivo Volf[1*]

[1]Institute of Physiology-Centre for Physiology & Pharmacology, Medical University of Vienna, Schwarzspanierstraße 17, 1090 Vienna, Austria

[2]Department of Otorhinolaryngology, Head and Neck Surgery, Medical University of Vienna, 1090 Vienna, Austria

*** Correspondence:** Corresponding Author: ivo.volf@meduniwien.ac.at

Supplementary Data: Questions used within VEX

The following pages include those questions that were used for VEX. Each question topic was tested according to competence levels $CL_{recognition}$, CL_{recall} and $CL_{concept}$.

Correct answers of depicted MC-questions are formatted in bold font.

For each question, percentage of correct answers in VEX is shown.

Contraction force ($SP_{objectives^{+}}$)

competence level		% correct
$CL_{concept}$	What mechanisms exist to increase the contractile force of a single muscle fiber?	38
CL_{recall}	What mechanisms are able to increase contractile force in skeletal muscle?	48
$CL_{recognition}$	What mechanisms are able to increase contractile force in skeletal muscle?<br><br>A increasing the number of motor end plates per muscle cell<br><br>**B recruitment of a greater number of motor units**<br><br>C enhancing removal of intracellular calcium<br><br>D prolongation of action potential duration<br><br>**E increasing frequency of action potentials**	53

Diastolic depolarisation ($SP_{objectives}^{+}$)

competence level		% correct
$CL_{concept}$	Describe the consequences of decreasing the slope of diastolic depolarization within the primary pacemaker of the heart.	37
CL_{recall}	Which part of the cardiac pacemaker / conduction system shows the steepest diastolic depolarization?	84
$CL_{recognition}$	Which part of the cardiac pacemaker / conduction system shows the steepest diastolic depolarization?<br><br>A Atrial myocardium<br><br>B **Sinus node**<br><br>C His bundle<br><br>D Purkinje fibers<br><br>E Ventricular myocardium	90

Cardiac valves ($SP_{objectives^+}$)

competence level		% correct
$CL_{concept}$	At a timepoint when the mitral valve is still open (final phase) and the aortic valve is closed: what pressure do you expect in the aorta (healthy person at rest, normal values)?	53
CL_{recall}	Which phase of the cardiac cycle is characterized by closed aortic valve and opened mitral valve?	87
$CL_{recognition}$	Which phase of the cardiac cycle is characterized by closed aortic valve and opened mitral valve?<br><br>A entire systole<br><br>B entire diastole<br><br>C ejection phase of systole<br><br>D isovolumetric contraction phase of systole<br><br>**E none of the above phases**	70

Lung volumes ($SP_{objectives^-}$)

competence level		% correct
$CL_{concept}$	Name the maximum volume you can exhale after complete (maximum) inhalation	53
CL_{recall}	The largest mobilizable (inhalable-exhalable) respiratory volume is called	80
$CL_{recognition}$	The largest of the listed respiratory volumes is the:<br><br>A Tidal volume at physical rest<br><br>B Inspiratory reserve volume<br><br>C Residual volume<br><br>**D Vital capacity**<br><br>E Expiratory reserve volume	97

Alveolar ventilation ($SP_{objectives^{+}}$)

competence level		% correct
$CL_{concept}$	What is the influence of hypoventilation on alveolar/arterial pCO_2? Please indicate a numerical value that is reached / exceeded / undercut	70
CL_{recall}	Alveolar pCO_2 in healthy individuals at sea level amounts to:	80
$CL_{recognition}$	Alveolar pCO_2 in healthy individuals at sea level amounts to :<br><br>A 10 mmHg<br>**B 40 mmHg**<br>C 100 mmHg<br>D 400 mmHg<br>E 760 mmHg	96

Pituitary gland ($SP_{objectives^-}$)

competence level		% correct
$CL_{concept}$	Describe the mode of action of ADH and a systemic consequence arising from ADH release	17
CL_{recall}	By which function does the pituitary gland regulate fluid balance?	59
$CL_{recognition}$	By which function does the pituitary gland regulate fluid balance?<br>A Production of ADH<br>B Production of oxytocin<br>**C Release of ADH in the posterior lobe**<br>D Release of oxytocin in the posterior lobe<br>E Triggering of thirst	39

Volume regulation ($SP_{objectives^-}$)

competence level		% correct
$CL_{concept}$	Increased stretching of the atria leads to a release of atriopeptin - what is the systemic consequence arising from ADH release?	53
CL_{recall}	The release of atriopeptin in the heart leads to?	68
$CL_{recognition}$	The release of atriopeptin in the heart leads to?<br><br>A Triggering of thirst<br>**B Increase in glomerular filtration rate**<br>C Release of aldosterone<br>D Release of ADH<br>E Increase in blood pressure	72

Kidney blood flow ($SP_{objectives}^{+}$)

competence level		% correct
$CL_{concept}$	What (cardio)vascular mechanisms may lead to decreased glomerular filtration rate?	36
CL_{recall}	The hydrostatic pressure within the glomerular capillaries and the associated glomerular filtration rate are kept fairly constant over wide ranges of blood pressure by autoregulation. What mechanisms lead to a decrease in hydrostatic pressure in the glomerular capillaries?	43
$CL_{recognition}$	The hydrostatic pressure within the glomerular capillaries and the associated glomerular filtration rate are kept fairly constant over wide ranges of blood pressure by autoregulation. What mechanisms lead to a decrease in hydrostatic pressure in the glomerular capillaries? (3 correct answers)<br><br>A Thirst<br><br>A Resistance increase in the efferent arterioles<br><br>**B Resistance increase in the afferent arterioles**<br><br>**C Vasoconstriction in the afferent arterioles**<br><br>D Vasoconstriction in the efferent arterioles<br><br>**E decrease in systemic mean blood pressure to values below 80 mmHg**	82

Glomerular filtration rate ($SP_{objectives^{+}}$)

competence level		% correct
$CL_{concept}$	What are the requirements for a substance you can use to determine the glomerular filtration rate?	23
CL_{recall}	A patient is injected with inulin to check his kidney function. What is the fate of inulin in the kidneys?	37
$CL_{recognition}$	A patient is injected with inulin to check his kidney function. What is the fate of inulin in the kidneys?<br><br>**A Filtration only**<br><br>B Filtration and reabsorption<br><br>C Filtration and secretion<br><br>D Resorption and secretion<br><br>E Filtration, resorption and secretion	84

Tendon organ ($SP_{objectives^-}$)

competence level		% correct
$CL_{concept}$	You perform resistance training in the gym - what protects your skeletal muscle from overload?	9
$CL_{concept}$	Describe the afferent neuron, central circuitry, and function of the Golgi tendon organ.	10
CL_{recall}	What stimulus does the tendon organ respond to?	48
$CL_{recognition}$	What stimulus does the tendon organ respond to?<br><br>A Pain<br><br>B Stretching of the joint capsule<br><br>C Inhibition of gamma motoneurons<br><br>**D Contraction of extrafusal muscle fibers**<br><br>E Contraction of intrafusal muscle fibers	73

Note: option "Contraction of extrafusal muscle fibers" ist not exactly correct, but was (also) accepted as a correct answer in CL_{recall}.

Regulation of osmolarity ($SP_{objectives}^{-}$)

competence level		% correct
$CL_{concept}$	Name mechanisms that are able to regulate increased plasma osmolarity	19
CL_{recall}	Which organs/tissues are involved in the regulation of plasma osmolarity?	79
$CL_{recognition}$	(3 correct answers)<br><br>**A Intestine**<br>B Spleen<br>**C Kidney**<br>**D Liver**<br>E Bone	85

Hyperkalemia ($SP_{objectives}^{-}$)

competence level		% correct
$CL_{concept}$	What consequence on cardiac function might arise from an increase in extracellular potassium concentration have?	17
CL_{recall}	An increase in potassium concentration in the extracellular space is called hyperkalemia. What are the implications for excitable cells?	69
$CL_{recognition}$	An increase in potassium concentration in the extracellular space is called hyperkalemia. What are the implications for excitable cells?<br><br>**A Nerve cells depolarize**<br><br>B Nerve cells hyperpolarize<br><br>C Membrane potential remains the same<br><br>D Calcium effluxes from the cell<br><br>E Additional potassium is released into the extracellular space	85

Renin – angiotensin system ($SP_{objectives}^{-}$)

competence level		% correct
$CL_{concept}$	Describe the basal effects of renin	16
CL_{recall}	Angiotensin II leads to:	76
$CL_{recognition}$	Angiotensin II leads to:<br><br>**A Sensation of thirst**<br><br>B Dilatation of arterioles<br><br>C Release of atriopeptin<br><br>D Inhibition of aldosterone release<br><br>E Drop in blood pressure	70

Menstrual cycle ($SP_{objectives^{+}}$)

competence level		% correct
$CL_{concept}$	Please draw the time course of estrogen and progesterone concentration during the menstrual cycle	32
$CL_{concept}$	Name the different phases of the menstrual cycle	66
CL_{recall}	What hormone increases during the luteal phase of the menstrual cycle?	90
$CL_{recognition}$	What hormone increases during the luteal phase of the menstrual cycle?<br><br>A Luteinizing hormone<br><br>B Follicle-stimulating hormone<br><br>C **Progesterone**<br><br>D ACTH<br><br>E Oxytocin	89

Synaptic transduction ($SP_{objectives}^{+}$)

competence level		% correct
$CL_{concept}$	Within the context of synaptic signal transduction: Describe the events occuring in the presynapse	79
CL_{recall}	What mechanism favors the triggering of an EPSP (excitatory postsynaptic potential)?	66
$CL_{recognition}$	What mechanism favors the triggering of an EPSP (excitatory postsynaptic potential)?<br><br>A Presynaptic efflux of calcium ions<br><br>**B Presynaptic influx of calcium ions**<br><br>C Presynaptic efflux of potassium ions<br><br>D Presynaptic influx of chloride ions<br><br>E Presynaptic efflux of sodium ions	93
